# Long-term administration of the mitochondria-targeted antioxidant mitoquinone mesylate fails to attenuate age-related oxidative damage or rescue the loss of muscle mass and function associated with aging of skeletal muscle

**DOI:** 10.1096/fj.201600450R

**Published:** 2016-08-22

**Authors:** Giorgos K. Sakellariou, Timothy Pearson, Adam P. Lightfoot, Gareth A. Nye, Nicola Wells, Ifigeneia I. Giakoumaki, Richard D. Griffiths, Anne McArdle, Malcolm J. Jackson

**Affiliations:** Medical Research Council–Arthritis Research UK Centre for Integrated Research into Musculoskeletal Ageing, Department of Musculoskeletal Biology, Institute of Ageing and Chronic Disease, University of Liverpool, Liverpool, United Kingdom

**Keywords:** heat shock protein, NF-κB, NOX4, SOD, superoxide

## Abstract

Age-related skeletal muscle dysfunction is the underlying cause of morbidity that affects up to half the population aged 80 and over. Considerable evidence indicates that oxidative damage and mitochondrial dysfunction contribute to the sarcopenic phenotype that occurs with aging. To examine this, we administered the mitochondria-targeted antioxidant mitoquinone mesylate {[10-(4,5-dimethoxy-2-methyl-3,6-dioxo-1,4-cyclohexadien-1-yl)decyl] triphenylphosphonium; 100 μM} to wild-type C57BL/6 mice for 15 wk (from 24 to 28 mo of age) and investigated the effects on age-related loss of muscle mass and function, changes in redox homeostasis, and mitochondrial organelle integrity and function. We found that mitoquinone mesylate treatment failed to prevent age-dependent loss of skeletal muscle mass associated with myofiber atrophy or alter a variety of *in situ* and *ex vivo* muscle function analyses, including maximum isometric tetanic force, decline in force after a tetanic fatiguing protocol, and single-fiber-specific force. We also found evidence that long-term mitoquinone mesylate administration did not reduce mitochondrial reactive oxygen species or induce significant changes in muscle redox homeostasis, as assessed by changes in 4-hydroxynonenal protein adducts, protein carbonyl content, protein nitration, and DNA damage determined by the content of 8-hydroxydeoxyguanosine. Mitochondrial membrane potential, abundance, and respiration assessed in permeabilized myofibers were not significantly altered in response to mitoquinone mesylate treatment. Collectively, these findings demonstrate that long-term mitochondria-targeted mitoquinone mesylate administration failed to attenuate age-related oxidative damage in skeletal muscle of old mice or provide any protective effect in the context of muscle aging.—Sakellariou, G. K., Pearson, T., Lightfoot, A. P., Nye, G. A., Wells, N., Giakoumaki, I. I., Griffiths, R. D., McArdle, A., Jackson, M. J. Long-term administration of the mitochondria-targeted antioxidant mitoquinone mesylate fails to attenuate age-related oxidative damage or rescue the loss of muscle mass and function associated with aging of skeletal muscle.

Age-related loss of muscle mass and function underlies morbidity and mortality that affects up to half the population aged 80 yr and older (1). Loss of muscle strength in the elderly is a contributor to loss of independence and to physical disability, which is linked to increased risk of falls and fractures. Many structural and functional changes occur with advancing age in skeletal muscle, including a reduction in the number and cross-sectional area (CSA) of individual muscle fibers (2).

Oxidative stress has been suggested to be among the factors contributing to the initiation and progression of sarcopenia that occurs during aging (3, 4). Reports from our group (5–11) and others (12, 13) have shown that genetic manipulations of redox regulatory systems modifies the muscle aging process. Skeletal muscle has a high content of mitochondria (14), and mitochondrial redox homeostasis has been proposed to play a key role in age-related oxidative damage (15). Consistent with a role of mitochondria as a contributor to age-related muscle redox changes, studies have shown that isolated skeletal muscle mitochondria exhibit an age-dependent increase in hydrogen peroxide (H_2_O_2_) generation (16, 17).

Reactive oxygen species (ROS) derived from mitochondria [mitochondrial ROS (mtROS)] are linked to the pathogenesis of a number of age-related human diseases, including neurodegenerative disorders, ischemia–reperfusion injury, and diabetes (18, 19). Considerable evidence has shown that mitochondrial oxidative damage can alter mitochondrial integrity and function in aging skeletal muscle, including a reduction in mitochondrial abundance (20) and oxidative phosphorylation (21), accumulation of mutated mitochondrial DNA (mtDNA) (15) associated with impaired mitophagy (22), and increased mitochondrial-mediated apoptosis (23), which could all contribute to sarcopenia. Although cumulative oxidative damage has been suggested to induce age-associated decline in mitochondrial function (24), the effects of mitochondrial dysfunction and mtROS in age-related muscle atrophy remains a controversial topic (25, 26).

To directly examine whether age-related atrophy and mitochondrial dysfunction is related to mitochondrial redox changes, we administered the mitochondria-targeted antioxidant mitoquinone mesylate {[10-(4,5-dimethoxy-2-methyl-3,6-dioxo-1,4-cyclohexadien-1-yl)decyl] triphenylphosphonium} to 24-mo-old for 15 wk and investigated the effects on age-related loss of muscle mass and function, changes in muscle redox homeostasis, and mitochondrial organelle function and content. We hypothesized that if alterations in the mitochondrial redox status are implicated in the processes of age-related muscle wasting, mitoquinone mesylate drug treatment would ameliorate the sarcopenic phenotype associated with loss of muscle mass and weakness. In the present study, we report the effects of long-term administration of mitoquinone mesylate on muscle mass, morphology, and function; redox homeostasis; adaptive responses; and mitochondrial integrity and function in aging skeletal muscle.

## MATERIALS AND METHODS

### Chemicals and reagents

Unless stated otherwise, all chemicals used in this study were obtained from Sigma-Aldrich (St. Louis, MO, USA).

### Mice

Male and female wild-type C57BL/6 mice (8 mo old) were obtained from Charles River Laboratories (Margate, United Kingdom) and aged to 28 mo at the Biomedical Services Unit, University of Liverpool. All experiments were conducted in accordance with UK Home Office guidelines under the UK Animals (Scientific Procedures) Act 1986. Mice were fed a CRM (P) rodent diet (Special Diet Services, Essex, United Kingdom) and were maintained under barrier conditions in microisolator cages on a 12-h dark/light cycle. For simple tissue collection, mice were humanely killed by cervical dislocation, and muscles and tissues were either rapidly removed, snap frozen, and stored at −80°C, or embedded in Tissue-Tek (VWR International, West Chester, PA, USA) and rapidly frozen in nitrogen-chilled isopentane for histologic analysis. Mice subjected to *in situ* muscle force measurements were anesthetized with intraperitoneal injections of ketamine hydrochloride (66 mg/kg) and medatomidine hydrochloride (0.55 mg/kg), with supplemental injections provided to maintain an adequate level of anesthesia throughout the procedure. All procedures were approved by the University of Liverpool Animal Welfare and Ethical Review Body.

### Mitoquinone mesylate administration

Mice were 24 mo of age at the start of the treatment and were administered 100 μM mitoquinone mesylate (as a β-cyclodextrin complex; Suzhou Vosun Chemical, Jiangsu, China) in their drinking water for the next 15 wk. Fresh mitoquinone mesylate solutions were provided twice a week; control mice were supplied with water without the supplement (*n* = 8 mice per group). All mice were monitored daily and weighed once a week. We selectively chose to administer mice with mitoquinone mesylate between the ages of 24 and 28 mo because preliminary studies showed that age-related muscle atrophy became apparent over the time period of 24 to 28 mo. The dose of mitoquinone mesylate used in the present study was based on that previously used (100 μM) to protect against oxidative damage in a mouse model of Alzheimer disease (27). Other previous studies have used mitoquinone mesylate doses as high as 500 μM for up to 28 wk (28).

### *In situ* muscle function analysis

Extensor digitorum longus (EDL) muscle contractile properties were measured *in situ* as previously described (5). To assess the maximum isometric tetanic force (*P*_o_) of the EDL muscle, the distal tendon from anesthetized mice was severed and secured to the lever arm of a servomotor (Aurora Scientific, Aurora, ON, Canada). The knee of the hind limb was fixed, the peroneal nerve was exposed, and bipolar platinum wire electrodes were placed across the nerve. Muscle optimal length (*L*_o_) was determined by variation of the length as the muscle was repeatedly stimulated at 1 Hz, and muscle length was set where maximum force was achieved. For determination of *P*_o_, EDL muscles were electrically stimulated to contract at *L*_o_ and optimal stimulation voltage (8–10 V) at 2-min intervals for 300 ms with 0.2-ms pulse width. *P*_o_ was assessed by increasing the frequency of stimulation from 10 to 50 Hz and subsequently in 50-Hz increments to a maximum of 300 Hz. *P*_o_ was identified when the maximum force reached a plateau despite increasing stimulation frequency. After identification of *P*_o_, mice were subjected to a repetitive tetanic fatiguing protocol, which consisted of 60 consecutive isometric contractions (300 ms at 100 Hz every 5 s for 5 min) (29). After completion of the procedures, mice were killed by cervical dislocation, and muscles and tissues were rapidly removed. Muscle fiber length (*L*_f_) and weight of EDL muscles were measured *ex vivo* to determine muscle CSA. Specific *P*_o_ (mN/mm^2^) was calculated by dividing *P*_o_ by total fiber CSA for each muscle.

### Determination of muscle structure

Anterior tibialis (AT) muscles were cryosectioned at −20°C through the midbelly with a thickness of 12 µm, and fluorescent immunohistochemical staining was undertaken on the same day. Sections were rinsed with PBS and permeabilized in 0.2% Triton X-100 in PBS for 5 min. Fluorescein-labeled wheat germ agglutinin (WGA; 5 µg/ml; Vector Laboratories, Burlingame, CA, USA) was used to identify extracellular matrix. Nuclei were identified using DAPI (1 µg/ml). Cross sections from 5 to 6 muscles per treatment group were examined by blinded observers to count the total number of fibers, percentage of centronucleated fibers, and individual fiber CSA. To ensure that all fibers per section were analyzed, consecutive images acquired from each cryosection at ×10 magnification were merged into a single high-resolution image using Adobe Photoshop CS5. ImageJ software (Image Processing and Analysis in Java; National Institutes of Health, Bethesda, MD, USA) was used to quantify individual fiber CSA.

### Confocal laser scanning microscopy

Fluorescence images were obtained using a C1 confocal laser scanning microscope (Nikon, Tokyo, Japan) equipped with a 405-nm excitation diode laser, a 488-nm excitation argon laser, and a 543-nm excitation helium–neon laser. Emission fluorescence was detected through a set of 450/35, 515/30, and 605/15 emission filters. Fluorescence images were analyzed by Nikon EZ-C1 3.9 (12 bit) acquisition software.

### Preparation of permeabilized muscle fiber bundles

Selective plasma membrane permeabilization of fiber bundles was performed according to methods described by Kuznetsov *et al.* (30) to allow analysis of intact skeletal muscle mitochondria *in situ* (19, 31). In brief, AT muscles were placed in ice-cold buffer A containing (mM) 50 K-MES, 7.23 K_2_EGTA, 2.77 CaK_2_EGTA, 20 imidazole, 0.5 DTT, 20 taurine, 5.3 Na_2_ATP, 15 PCr, and 6.56 MgCl_2_ · 6H_2_O (pH 7.3 at 4°C) and trimmed of connective tissue and fat. Muscles were manually teased into small bundles of fibers and treated with 50 μg/ml saponin (in buffer A) for 30 min at low rocking speed. After permeabilization, fiber bundles prepared for mitochondrial H_2_O_2_ emission measurements were washed 3 × 10 min in ice-cold buffer Z containing (mM) 110 K-MES, 35 KCl, 1 EGTA, 5.3 Na_2_ATP, 10 K_2_HPO_4_, and 3 MgCl_2_ · 6H_2_O (pH 7.3 at 4°C), supplemented with 5 mg/ml bovine serum albumin. Permeabilized fiber bundles prepared for respiration analyses were washed 3 × 10 min in ice-cold buffer B containing (mM) 100 K-MES, 7.23 K_2_EGTA, 2.77 CaK_2_EGTA, 20 imidazole, 0.5 DTT, 20 taurine, 3 K_2_HPO_4_, and 1.38 MgCl_2_ · 6H_2_O (pH 7.3 at 4°C) supplemented with 2 mg/ml bovine serum albumin.

### *Ex vivo* single muscle fiber analysis

Isolated intact single muscle fibers were excised from the AT muscle and maintained in ice-cold relax solution containing (mM) 4.5 MgATP, 1 free Mg^2+^, 10 imidazole, 2 EGTA, and 100 KCl (pH 7.0) (32). Single fibers were permeabilized with 50 μg/ml saponin (in relax solution) for 15 min on ice. Permeabilized fibers were mounted on an 802D muscle testing apparatus (Aurora Scientific), mounted on insect pins with fine thread, and attached to a 403A (5-mN) force transducer and 312C length controller. Single fibers were maximally activated (pCa 4.5) (−log[free Ca^2+^]) containing (mM) 5.3 MgATP, 1 free Mg^2+^, 20 imidazole, 7 EGTA, 19.6 PCr, and 64 KCl (pH 7.0). Maximal force was recorded for each fiber and normalized to CSA (32). Sarcomere length for each individual myofiber was adjusted to 2.4 to 2.6 μm; the diameter was measured at 4 intervals along the length of the fiber, and circular circumference was assumed for the calculation of CSA.

### Mitochondrial respiration analysis

Permeabilized myofiber respiration was assessed using a Clark-type electrode in a continuously stirred sealed and thermostatically controlled chamber (Oxytherm System; Hansatech Instruments, King’s Lynn, United Kingdom) maintained at 37°C. After calibration of the respiration chamber, permeabilized bundles (∼15 mg wet weight) were incubated in respiration buffer B (22, 31). Respiration (O_2_ consumption) was determined using glutamate (5 mM) and malate (5 mM) substrates. ADP-stimulated respiration (state 3) was initiated by addition of ADP (0.3 mM). Respiratory control index (RCI) was calculated by dividing state 3 by state 4 respiration, and the efficiency of oxidative phosphorylation was determined by calculating the ratio of ATP amount to consumed O_2_ during state 3 respiration (P:O ratio).

### Mitochondrial H_2_O_2_ emission measurements

Mitochondrial H_2_O_2_ efflux was measured using the Amplex Red–horseradish peroxidase (Molecular Probes, Eugene, OR, USA) assay as previously described (16). H_2_O_2_ production was expressed as picomoles per minute per unit of citrate synthase (CS) activity.

### mtDNA quantification

mtDNA was measured by real-time quantitative RT-PCR (qRT-PCR) as described by Chen *et al.* (33).

### DNA damage and DNA fragmentation

DNA damage was assessed by the content of 8-hydroxydeoxyguanosine (8-OHdG) as described by Changou *et al.* (34). Apoptotic DNA fragmentation was assessed by DNA laddering using agarose gel electrophoresis as described by Houot *et al.* (35).

### Fluorescence-based methods to measure mitochondrial membrane potential and MitoSOX Red oxidation

To monitor changes in mitochondrial superoxide, isolated permeabilized fibers from the AT muscle were loaded with MitoSOX Red (250 nM, Thermo Fisher Scientific, Waltham, MA, USA) for 30 min as previously described (36). Fibers were maintained in Z buffer containing MitoSox Red (20 nM) during the experimental period. The reaction between superoxide and MitoSox Red generates a specific red fluorescent product, 2-hydroxyethidium (2-OH-Mito-E^+^) (37), monitored at an excitation/emission wavelength of 405/605 nm. Measurement of mitochondrial membrane potential (ΔΨ_m_) in intact mitochondria of isolated AT fibers was assessed by tetramethylrhodamine, methyl ester (TMRM, 30 nM; Thermo Fisher Scientific) fluorescence at an excitation/emission wavelength of 543/605 nm. Changes in ΔΨ_m_ were determined in the presence of the oxidative phosphorylation inhibitors oligomycin (2.5 μM) and protonophore carbonylcyanide-p-trifluoromethoxyphenyl hydrazone (FCCP; 4 μM).

### Enzymatic activity assays

Enzymatic activity of CuZnSOD and MnSOD was assessed in native gels, with negative staining, as described previously (6, 8). Aconitase activity was quantified by measuring the reduction of NADP^+^ to NADPH after addition of 2 U of isocitrate dehydrogenase by using a microplate fluorometer (FluoStar Optima, BMG Labtech; Thermo Fisher Scientific) at an excitation/emission wavelength of 360/460 nm (38). Mitochondrial enzyme CS activity was determined spectrophotometrically using the MitoCheck Citrate Synthase Activity Assay (Cayman Chemicals, Ann Arbor, MI, USA) according to manufacturer’s protocol. Respiratory chain complex I activity in skeletal muscle homogenates was examined by the reduction of 2,6-dichloroindophenol, followed spectrophotometrically at 600 nm as described by Janssen *et al.* (39).

### Real-time qRT-PCR analysis

RNA from skeletal muscle was extracted, DNase treated, and purified using Direct-zol RNA miniprep (Zymo Research, Irvine, CA, USA). Purified RNA was utilized to generate first-strand cDNA using the iScript cDNA Synthesis kit (Bio-Rad, Hercules, CA, USA). Primers for real-time qRT-PCR analyses are shown in **Table 1**; and the optimal annealing temperature for each primer set was determined by using an annealing temperature gradient between 55 and 62°C. Real-time PCR reactions were performed on an iCycler Detection System (Bio-Rad) using iQ SYBR Green Supermix (Bio-Rad). Specificity of the PCR products was determined by melt curve analysis and agarose gel electrophoresis. Three reference genes—glyceraldehyde 3-phosphate dehydrogenase (GAPDH), β-2 microglobulin (B2M), and ribosomal protein S29 (RPS29)—were used as internal controls.

**TABLE 1.** Sequences of specific primers used for real-time qRT-PCR amplification

Name	Primer sequence, 5′–3′		Amplicon size (bp)
	Forward	Reverse	
GAPDH	CCGTAGACAAAATGGTGAAGG	TCGTTGATGGCAACAATCTC	109
B2M	GGAGAATGGGAAGCCGAACA	TCTCGATCCCAGTAGACGGT	249
RPS29	ATGGGTCACCAGCAGCTCTA	GTATTTGCGGATCAGACCGT	102
COX I	CACTAATAATCGGAGCCCCA	TTCATCCTGTTCCTGCTCCT	129
COX IV	TGGGAGTGTTGTGAAGAGTGA	GCAGTGAAGCCGATGAAGAAC	273
CS	CAAGATTGTGCCCAATATCCTC	TTCATCTCCGTCATGCCATA	111
MCIP1	CAGCGAAAGTGAGACCAGGG	ACGGGGGTGGCATCTTCTAC	309
TFAM	GCTGATGGGTATGGAGAAG	GAGCCGAATCATCCTTTGC	161
PGC-1α	TTCCACCAAGAGCAAGTAT	CGCTGTCCCATGAGGTATT	131
NRF1	TTACTCTGCTGTGGCTGATGG	CCTCTGATGCTTGCGTCGTCT	92
OPA1	TCAGCAAAGCTTACATGCAGA	TGCTTGGACTGGCTACATTTT	180
MFN1	TGCCCTCTTGAGAGATGACC	AGAGCCGCTCATTCACCTTA	182
MFN2	GGGGCCTACATCCAAGAGAG	CCTTGGACAGGTACCCTTTG	115
FIS1	GCCTGGTTCGAAGCAAATAC	CACGGCCAGGTAGAAGACAT	116
DRP1	CTGACGCTTGTGGATTTACC	CCCTTCCCATCAATACATCC	277
ND1	CCTATCACCCTTGCCATCAT	GAGGCTGTTGCTTGTGTGAC	194
PECAM 1	ATGGAAAGCCTGCCATCATG	TCCTTGTTGTTCAGCATCAC	235

COX I, cytochrome *c* oxidase subunit I; COX IV, cytochrome *c* oxidase subunit IV; DRP1, dynamin-related protein 1; FIS1, mitochondrial fission 1 protein; MCIP1, modulatory calcineurin interacting protein 1; MFN1, mitofusin 1; MFN2, mitofusin 2; ND1, mitochondrial encoded NADH dehydrogenase 1; NRF1, nuclear respiratory factor 1; OPA1, optic atrophy type 1; PECAM 1, platelet endothelial cell adhesion molecule 1; TFAM, mitochondrial transcription factor A.

### Immunoblotting

Protein extracts (20 µg per sample) were separated using a standard protocol for Western blot analysis (7). Peroxidase activity was detected using an ECL kit (Amersham Pharmacia Biotech, Piscataway, NJ, USA), and band intensities were analyzed using Quantity One software (Bio-Rad). Mitochondrial and cytosolic subcellular fractions were obtained from skeletal muscle as previously described (40).

### Statistical analyses

Data are presented as means ± sem for each experiment. Statistical analyses for potential differences between groups were determined by ANOVA followed by the *post hoc* least significant difference test. Single comparisons between 2 experimental conditions were undertaken by the unpaired Student’s *t* test. Data were analyzed by SPSS 22 (IBM SPSS, Chicago, IL, USA), and values of *P* < 0.05 were considered statistically significant.

## RESULTS

### Age-related loss of muscle mass is associated with myofiber atrophy

**Figure 1.**
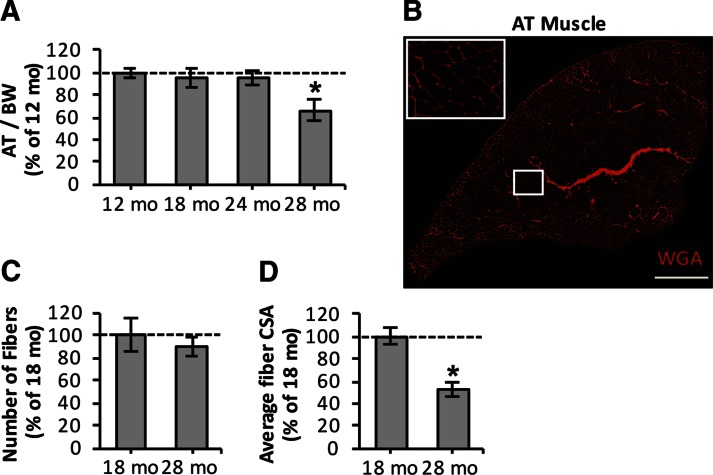
Sarcopenia is associated with myofiber atrophy. *A*) Age-related changes in AT muscle mass, normalized to BW. *P < 0.05 compared to values from other age groups (*n* = 6–8 mice per group). *B*) Transverse section of AT muscle from 18-mo-old mouse stained with WGA (5 μg/ml, red), to visualize extracellular matrix and to assess total fiber numbers and CSA of individual fibers. Scale bar, 500 μm. *C*) Number of muscle fibers in AT muscle from 18- and 28-mo-old mice. *D*) Average fiber CSA of individual fibers from AT muscle of 18- and 28-mo-old mice. *P < 0.05 compared to values from 18-mo-old mice (*n* = 6–8 mice per group).

To determine the time course of age-related phenotypic changes that occur in skeletal muscle, we initially assessed AT muscle mass at 12, 18, 24, and 28 mo of age in mice (**Fig. 1*A***). AT muscle showed age-related loss of muscle mass [∼34% reduction in mass relative to body weight (BM)] at 28 mo of age (Fig. 1*A*). To understand the structural muscle changes underlying sarcopenia, immunohistochemical analysis of AT muscle (Fig. 1*B*) was undertaken for 18- and 28-mo-old mice. The total number of fibers per AT muscle tended to be reduced by a mean of ∼11% in 28-mo-old mice compared to 18-mo-old mice (Fig. 1*C*), but the changes were not significantly different. We then examined whether AT muscle of 28-mo-old mice showed atrophy of the remaining muscle fibers (Fig. 1*D*). Average muscle fiber CSA was reduced significantly by a mean ∼46% from 18 to 28 mo of age, suggesting that myofiber atrophy is the predominant cause of the loss of skeletal muscle mass that occurs between these ages.

### Long-term administration of mitochondria-targeted antioxidant mitoquinone mesylate fails to attenuate age-related mtROS increase in muscle fibers from old mice

**Figure 2.**
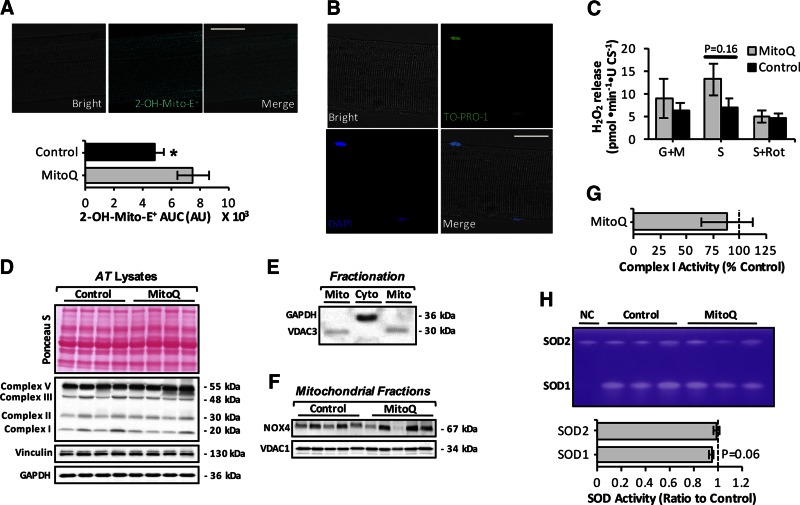
Effect of long-term mitoquinone mesylate (MitoQ) administration on mtROS in skeletal muscle of old mice. *A*) Upper panels: representative images of single fiber isolated from AT muscle under bright-field fluorescent image after loading with MitoSOX Red (20 nM, cyan) and merged image as indicated and analyzed by confocal microscopy. Original magnification, ×60. Scale bar, 25 μm. Lower panel: statistical analysis of area under mitochondrial 2-OH-Mito-E^+^ fluorescence trace (area under curve) over 60 min for control old mice and mice treated with MitoQ for 15 wk (24–28 mo old), arbitrary units (AU). 2-OH-Mito-E^+^ fluorescence was normalized to CS activity. *P < 0.05 compared to values from MitoQ-treated old mice (*n* = 12 fibers, 5–6 mice per group). *B*) Confocal images of saponin-permeabilized fiber isolated from AT muscle under bright-field fluorescent image after loading with To-Pro-1 iodide (200 nM, green) and DAPI (1 μg/ml, blue); merged image as indicated and analyzed by fluorescence microscopy. Original magnification, ×60. Scale bar, 25 μm. *C*) Mitochondrial H_2_O_2_ production (normalized per CS activity) assessed in permeabilized fiber bundles prepared from AT muscle of control and MitoQ-treated old mice. Mitochondrial substrates and inhibitors—glutamate and malate (G/M; 5 mM for both), succinate (S; 10 mM), and rotenone (Rot; 1 μM)—were added as indicated (*n* = 5–6 mice per group). *D*) Protein levels of oxidative phosphorylation (oxphos) complexes (I, II, III, and V) from AT muscle of control and MitoQ-treated old mice. Intensity of bands shown in Ponceau S–stained gel (upper) was equivalent to GAPDH and vinculin protein levels (lower) and were used as loading controls. *E*) Representative Western blot of GAPDH and voltage-dependent anion channel 3 (VDAC3) content to illustrate purity of extracted mitochondrial (Mito) and cytosolic (Cyto) skeletal muscle fractions. *F*) Protein levels of NOX4 in skeletal muscle mitochondrial fractions of control and MitoQ-treated old mice. *G*) Rotenone-sensitive respiratory chain complex I activity in AT skeletal muscle homogenates of control and MitoQ-treated old mice (*n* = 5–6 mice per group). *H*) Native gels stained for SOD1 and SOD2 enzyme activities in AT skeletal muscle of control and MitoQ-treated old mice (upper) and densitometric quantification of bands (lower). Negative control (NC) included AT muscle lysate from Sod1-null mice.

Previous studies from our group (16) and others (17) have shown that isolated skeletal muscle mitochondria exhibit an age-related increase in H_2_O_2_, which is associated with increased mitochondrial oxidative damage (21). To assess whether age-dependent mtROS increase and oxidative damage are contributing factors to the loss of muscle mass that occurs with aging, 24-mo-old mice were treated with mitochondria-targeted mitoquinone mesylate for 15 wk. We chose to administer the compound to animals between the ages of 24 and 28 mo because our data (Fig. 1*A*) revealed that loss of muscle mass occurs after 24 mo of age. To assess the antioxidant effect of mitoquinone mesylate, single muscle fibers isolated from the AT muscle of control and mitoquinone mesylate-treated old mice were loaded with MitoSOX Red (**Fig. 2*A***, upper). mitoquinone mesylate treatment was found to increase the 2-OH-Mito-E^+^ fluorescence (Fig. 2*A*, lower), indicating a potential increase in mitochondrial superoxide production. To further assess changes in mtROS in the presence of mitochondrial substrates and inhibitors, we determined H_2_O_2_ efflux from intact mitochondria in permeabilized myofibers from the AT muscle (Fig. 2*B, C*). Saponin-permeabilized fibers from the AT muscle displayed good morphology and well-defined striations along the sarcolemma and stained positive for To-Pro-1 iodide (Thermo Fisher Scientific), indicating plasma membrane permeabilization (Fig. 2*B*). Glutamate/malate (complex I substrates) and succinate (complex II substrate) fueled mitochondria from treated old mice showed a tendency toward a higher increase in H_2_O_2_ emission compared to control old mice (Fig. 2*C*) but did not reach statistical significance. The succinate induced H_2_O_2_ release was abolished by the complex I inhibitor rotenone, suggesting that this H_2_O_2_ release likely derives from complex I (Fig. 2*C*).

Because these results were unexpected, we next sought to determine whether long-term administration of mitoquinone mesylate altered the expression of potential sources for mtROS generation, including complexes I, II, and III (41) and mitochondrial nicotinamide adenine dinucleotide phosphate oxidase 4 (NOX4) (14, 36). To assess mitochondrial protein levels of NOX4, mitochondrial and cytosolic fractions from skeletal muscle of control and mitoquinone mesylate-treated old mice were prepared (Fig. 2*E*). We observed no changes in protein expression of complexes I, II, and III (Fig. 2*D*) or mtNOX4 (Fig. 2*F*). Similarly, no changes were observed in the enzymatic activities of respiratory complex I (Fig. 2*G*) or mitochondrial matrix superoxide dismutase (SOD) 2 (Fig. 2*H*). A tendency to a reduction in SOD1 activity was observed in skeletal muscle of mitoquinone mesylate-treated old mice compared to control old mice (*P* = 0.06). These findings were somewhat surprising but indicate that long-term mitoquinone mesylate treatment of old mice failed to attenuate the age-related increase in ROS generation by intact mitochondria in isolated skeletal muscle fibers.

### Mitoquinone mesylate fails to attenuate oxidative damage in aging skeletal muscle

**Figure 3.**
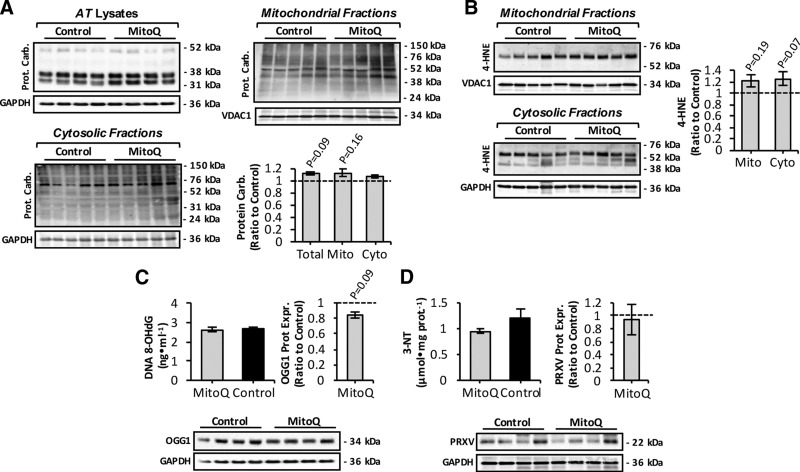
Markers of oxidative damage in skeletal muscle from mitoquinone mesylate (MitoQ)-treated old mice. *A*) Western blot analysis and quantification (lower right) of protein carbonyls in mitochondrial (upper right) and cytosolic (lower left) skeletal muscle fractions, and AT lysates (upper left) of control and MitoQ-treated old mice. *B*) Western blot analysis (left) and quantification (right) of 4-HNE protein adducts in mitochondrial (upper left) and cytosolic skeletal muscle fractions (lower left) of control and MitoQ-treated old mice. *C*) Levels of 8-OHdG in genomic DNA extracted from skeletal muscle (upper left), and OGG1 protein levels (lower) of skeletal muscle from control and MitoQ-treated old mice and densitometric quantification of blot (*n* = 5–6 mice per group; upper right). *D*) 3-NT content (upper left) and PRXV protein levels (lower) of skeletal muscle from control and MitoQ-treated old mice and densitometric quantification of blot (*n* = 5–6 mice per group; upper right).

To determine whether long-term mitoquinone mesylate treatment altered levels of age-related oxidative damage in skeletal muscle (42–44), we next examined the amounts of protein oxidation, lipid peroxidation, DNA damage, and protein nitration (**Fig. 3**). Mitoquinone mesylate treatment tended to increase age-related protein carbonylation (9) in whole AT muscle (Fig. 3*A*, upper left) and mitochondrial fractions (Fig. 3*A*, upper right). There was also a tendency for an increase in protein carbonyl content of cytosolic fractions from mitoquinone mesylate-treated mice (Fig. 3*A*, lower). Assessment of lipid peroxidation in mitochondrial and cytosolic fractions from skeletal muscle of control and mitoquinone mesylate-treated old mice was undertaken by immunoblotting for 4-hydroxynonenal (4-HNE) protein adducts (Fig. 3*B*). The data obtained were similar to those for protein oxidation in that mitoquinone mesylate treatment tended to increase lipid peroxidation in both fractions (Fig. 3*B*). However, neither protein carbonylation nor lipid peroxidation markers showed statistically significant differences between control and mitoquinone mesylate-treated mice. The extent of oxidative DNA damage was also assessed by examining 8-OHdG in genomic DNA and protein expression of oxoguanine DNA glycosylase (OGG1), a primary enzyme responsible for the excision of 7,8-dihydro-8-oxoguanine lesion (45) (Fig. 3*C*). Muscle from mitoquinone mesylate-treated old mice showed a trend toward a reduction (*P* = 0.09) in OGG1 protein levels (Fig. 3*C*, right and lower), with no change in the levels of 8-OHdG (Fig. 3*C*, left). Finally, the level of protein nitration (3-nitrotyrosine, 3-NT) and the expression of peroxiredoxin V (PRXV), a peroxynitrite reductase (7), were determined (Fig. 3*D*), but no effects on 3-NT content (Fig. 3*D*, left) or PRXV expression (Fig. 3*D*, right and lower) were observed. Overall, these data indicate that long-term administration of mitoquinone mesylate did not reduce oxidative damage in muscle of old mice.

### Mitoquinone mesylate alters expression of redox regulatory proteins in aging skeletal muscle

**Figure 4.**
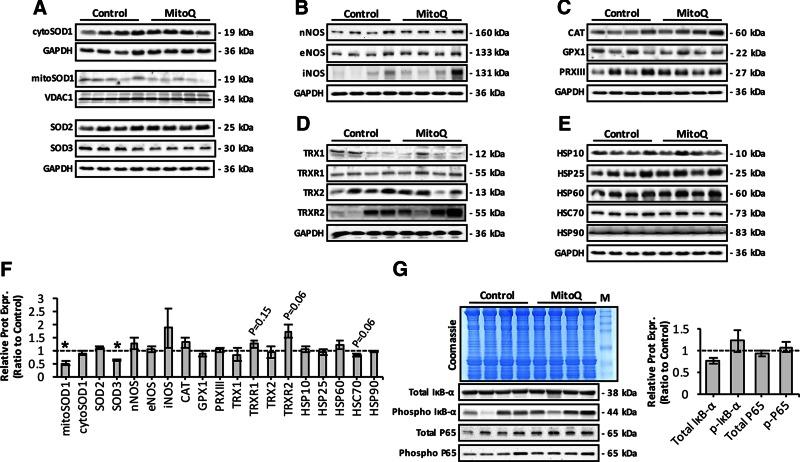
Effect of long-term mitoquinone mesylate (MitoQ) treatment on RONS regulatory protein expression in skeletal muscle of old mice. *A*) Representative Western blots depicting SOD isoform expression in AT lysates and mitochondrial/cytosolic skeletal muscle fractions of control and MitoQ-treated old mice. *B*) Protein expression levels of NOS isoforms in AT lysates of control and MitoQ-treated old mice. *C*) Western blots of main H_2_O_2_-reducing enzymes, including catalase (CAT), glutathione peroxidase 1 (GPX1), and PRXIII, in AT lysates of control and MitoQ-treated old mice. *D*) Protein expression of main redox proteins involved in TRX-PRX system, including TRX1, TRX2, TRXR1, and TRXR2, in AT lysates of control and MitoQ-treated old mice. *E*) Western blots of heat shock proteins in AT lysates of control and MitoQ-treated old mice. *F*) Densitometric analysis of represented Western blots shown in *A–E*. *P < 0.05 compared to values from old control mice. *G*) Effect of long-term MitoQ treatment on total and phosphorylated IκB-α (phospho IκB-α) and P65 content (total and phosphorylated) (lower left), and densitometric quantification of blots (right). Coomassie Brilliant Blue–stained gel (upper left) served as loading control. M, molecular weight marker.

To determine whether long-term mitoquinone mesylate treatment of old mice caused adaptations in the expression of proteins involved in antioxidant defense, we measured the expression of redox regulatory proteins, including SOD isoforms (**Fig. 4*A***); NOS isoenzymes (Fig. 4*B*); H_2_O_2_-scavenging enzymes, including glutathione peroxidase 1, catalase, and PRXIII (Fig. 4*C*); redox proteins involved in the thioredoxin–peroxiredoxin (TRX-PRX) system (Fig. 4*D*); and heat shock proteins (Fig. 4*E*), which have all been shown to provide protection against the damaging effects of increased reactive oxygen and nitrogen species (RONS) production (6, 9). Densitometric quantification (Fig. 4*F*) of the blots presented in Fig. 4*A–E* revealed a significant reduction in protein expression of extracellular SOD3 isoform and mitochondrial SOD1. This was also associated with a trend toward increased levels of TRX-PRX regulatory proteins including thioredoxin reductase 1 (TRXR1), mitochondrial TRXR2, and iNOS and a reduction in HSC70 (Fig. 4*F*). The NF-κB signaling pathway is known to regulate the expression of iNOS (46) and ROS antioxidant enzymes (4), and previous *in vitro* studies have demonstrated enhanced NF-κB activation after incubation with mitoquinone mesylate (47). We therefore examined activation of the NF-κB pathway, which we previously demonstrated to be increased in skeletal muscle of old mice (48). Muscle of mitoquinone mesylate-treated old mice showed no significant change in activation of the NF-κB pathway as indicated by phosphorylation of IκB-α, total IκB-α content (a key inhibitor of NF-κB activation), or NF-κB-P65 protein content (total and phosphorylated) (Fig. 4*G*). Overall, these data suggest that long-term treatment of mitoquinone mesylate may have altered the expression of a small number of specific redox regulatory proteins in skeletal muscle from old mice.

### Long-term mitoquinone mesylate treatment does not affect mitochondrial abundance in skeletal muscle of old mice

**Figure 5.**
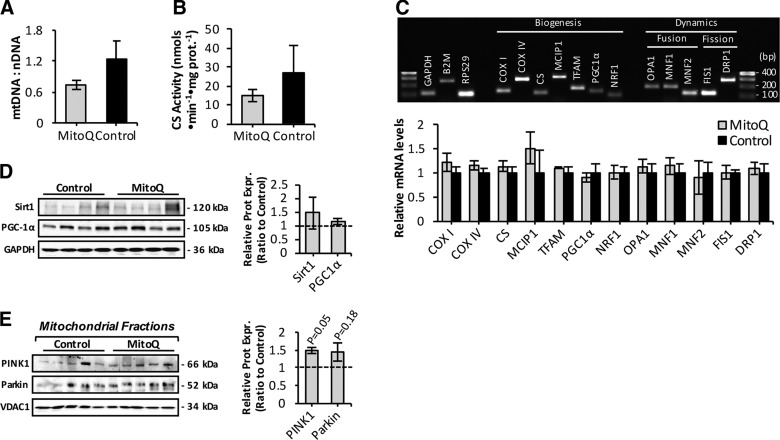
Effect of long-term mitoquinone mesylate (MitoQ) administration on mitochondrial content and mitophagy in skeletal muscle of old mice. *A*) Real-time qRT-PCR measurement of mtDNA, normalized to amount of nuclear DNA (nDNA) in skeletal muscle of control and MitoQ-treated old mice (*n* = 5–6 mice per group). *B*) CS activity in skeletal muscle of control and MitoQ-treated old mice (*n* = 5–6 mice per group). *C*) Representative image of agarose gel electrophoresis of real-time RT-PCR amplification products of GAPDH, B2M, RPS29, COXI, COXIV, CS, MCIP1, mitochondrial transcription factor A (TFAM), PGC-1α, nuclear respiratory factor 1 (NRF1), OPA1, MNF1, MNF2, FIS1, and DRP1 transcripts (upper). Lanes 1 and 17, 100 bp DNA molecular weight marker. PCR products correspond to amplicon sizes listed in Table 1. Relative mRNA levels of genes involved in mitochondrial biogenesis and dynamics analyzed by real-time qRT-PCR (lower). mRNA levels were normalized against the housekeeping genes GAPDH, B2M, and RPS29. *D*) Protein expression of sirtuin 1 (Sirt1) and PGC-1α mitochondrial biogenesis regulators (left) in AT skeletal muscle of control and MitoQ-treated old mice and densitometric quantification of blots (right). *E*) Western blots of isolated mitochondrial fractions from skeletal muscle of control and MitoQ-treated old mice immunodetected for PINK1, and ubiquitin ligase Parkin, mitophagy markers (left), and densitometric quantification of blots (right).

We sought to determine whether mitoquinone mesylate-treated old mice showed a change in mitochondrial abundance (15, 20) (**Fig. 5*A, B***). Muscle of treated old mice showed a tendency to a reduction in mtDNA copy numbers per nuclear genome (Fig. 5*A*) and CS activity (Fig. 5*B*). Real-time qRT-PCR analysis of expression of genes involved in mitochondrial dynamics (fusion and fission) and biogenesis (Fig. 5*C*, upper) showed that mRNA levels were similar between muscle of mitoquinone mesylate-treated and control old mice (Fig. 5*C*, lower). Protein levels of the transcriptional coactivator and regulator of mitochondrial biogenesis peroxisome proliferator-activated receptor α (PGC-1α) did not differ between control and mitoquinone mesylate-treated old mice (Fig. 5*D*). A potential effect on mitophagy was investigated using isolated mitochondrial fractions from skeletal muscle of control and mitoquinone mesylate-treated old mice immunoblotted for PTEN-induced putative kinase 1 (PINK1), and ubiquitin ligase Parkin mitophagy markers (Fig. 5*E*). We observed increased recruitment of PINK1 but no significant difference in Parkin in isolated mitochondria from mitoquinone mesylate-treated old mice (Fig. 5*E*) compared to values from control old mice, suggesting an increased mitophagic response. Levels of sirtuin 1 (Fig. 5*D*), an important regulator of mitochondrial recycling through the process of autophagy (49), were unchanged in the treated mice.

### Long-term mitoquinone mesylate treatment does not influence mitochondrial membrane potential in intact mitochondria of permeabilized muscle fibers in old mice

**Figure 6.**
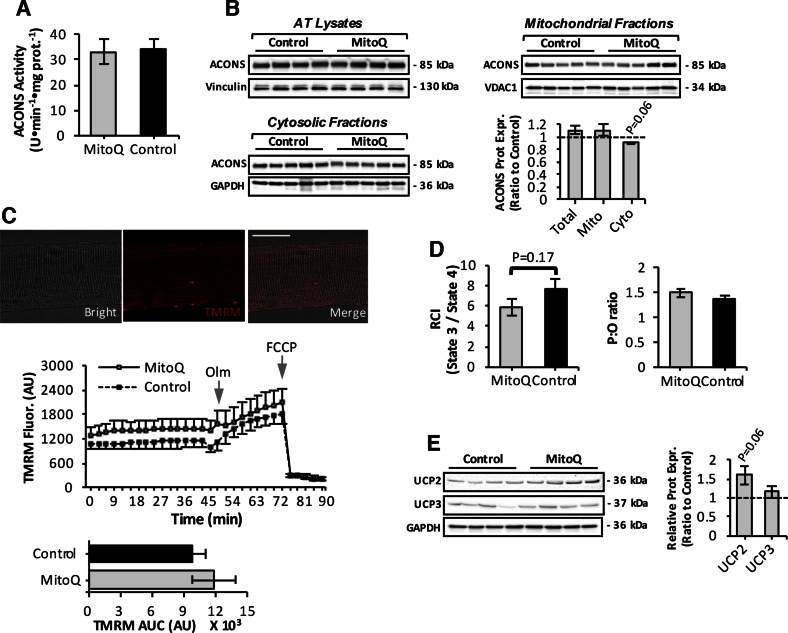
Mitochondrial function in skeletal muscle of mitoquinone mesylate (MitoQ)-treated old mice. *A*) Mitochondrial aconitase (ACONS) activity in AT skeletal muscle of 28-mo-old control and MitoQ-treated mice (*n* = 5–6 mice per group). *B*) Western blot analysis and quantification (lower right) of ACONS in mitochondrial (upper right) and cytosolic (lower left) skeletal muscle fractions, and AT lysates (upper left) of control and MitoQ-treated old mice. *C*) Confocal images of single fiber isolated from AT muscle under bright-field fluorescent image after loading with TMRM fluorescence (20 nM, red); merged image as indicated and analyzed by fluorescence microscopy. Original magnification, ×60. Scale bar, 30 μm (upper); Measurement of ΔΨ_m_ in intact mitochondria of isolated AT fibers from control and MitoQ-treated old mice, assessed by changes in TMRM fluorescence in response to oligomycin (Olm; 2.5 μM) and FCCP (4 μM), added at indicated time points (center); statistical analysis of area under TMRM fluorescence trace (area under curve) for control and MitoQ-treated old mice (*n* = 10–12 fibers, 5–6 mice per group; lower). *D*) Respiratory function of intact mitochondria in saponin-permeabilized myofibers from control and MitoQ-treated old mice shown by changes in RCI (left) and ratio of ATP amount to consumed O_2_ during state 3 (P:O ratio) (right) (*n* = 5–6 mice per group). *E*) UCP2 and UCP3 protein levels in skeletal muscle of control and MitoQ-treated old mice (left) and densitometric quantification of blots (right).

Cumulative oxidative damage has been proposed to induce age-associated reductions in mitochondrial function (24, 50). We thus we investigated mitochondrial function in skeletal muscle of mitoquinone mesylate-treated old mice (**Fig. 6**). Skeletal muscle of mitoquinone mesylate-treated old mice exhibited similar levels of mitochondrial aconitase activity (Fig. 6*A*) and mitochondrial protein levels (Fig. 6*B*, upper right) compared to control old mice, although a tendency to a reduction in aconitase content (*P* = 0.06) was observed in the cytosolic compartment of the AT muscle (Fig. 6*B*, lower left). The ΔΨ_m_ of intact mitochondria in isolated AT muscle fibers was examined by changes in TMRM fluorescence (Fig. 6*C*, upper) after treatment with oligomycin and the protonophore FCCP (Fig. 6*C*, center). Statistical analysis of the area under the TMRM fluorescence trace revealed differences between mitoquinone mesylate-treated and control old mice (Fig. 6*C*, lower). Next, we evaluated mitochondrial respiratory function in saponin-permeabilized fiber bundles after addition of glutamate/malate substrates and ADP. Myofibers from mitoquinone mesylate-administered old mice showed a trend toward a reduction (*P* = 0.17) in RCI (Fig. 6*D*, left) associated with a trend toward increased uncoupling protein (UCP) 2 protein levels (*P* = 0.06) (Fig. 6*E*). No changes in P:O ratio (Fig. 6*D*, right) or UCP3 protein expression (Fig. 6*E*) were observed in mitoquinone mesylate-treated old mice compared to control old mice.

### Mitochondrial-mediated apoptosis is not altered in response to long-term mitoquinone mesylate treatment in aging skeletal muscle

**Figure 7.**
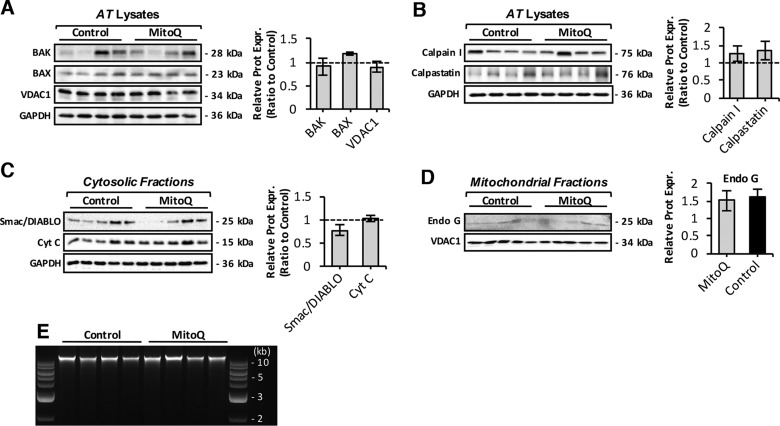
Effect of long-term mitoquinone mesylate (MitoQ) treatment on mitochondrial-mediated apoptosis in skeletal muscle. *A*) Immunoblots of BAK, BAX, and VDAC1 proapoptotic proteins in skeletal muscle of control and MitoQ-treated old mice (left) and densitometric quantification of blots (right). *B*) Protein expression levels of calpain I and calpastatin proteolytic enzymes in muscle of control and MitoQ-treated old mice (left) and densitometric quantification of blots (right). *C*) Western blots of isolated cytosolic fractions of control and MitoQ-treated mice immunodetected for cytochrome *c* (Cyt *C*) and Smac/DIABLO mitochondrial proapoptotic proteins (left), and densitometric quantification of blots (right). *D*) Protein levels of proapoptotic factor endonuclease G (Endo G) in skeletal muscle mitochondrial fractions of control and MitoQ-treated old mice (left) and densitometric quantification of blot (right). *E*) DNA fragmentation of genomic DNA isolated from skeletal muscle of control and MitoQ-treated old mice analyzed by agarose-gel electrophoresis. Lanes 1 and 10, 1 kb DNA molecular weight marker.

A variety of proapoptotic markers were examined, including expression of BAK, BAX, and VDAC1 (**Fig. 7*A***), proteolytic enzymes linked to apoptosis including expression of calpain I and calpastatin (Fig. 7*B*), mitochondrial release of cytochrome *c* and Smac/DIABLO proapoptotic proteins to cytosol (Fig. 7*C*), mitochondrial endonuclease G (Fig. 7*D*), and DNA fragmentation of genomic DNA (Fig. 7*E*). These results showed no changes in mitochondrial-mediated apoptotic processes in response to mitoquinone mesylate treatment.

### Long-term administration of mitoquinone mesylate failed to prevent loss of muscle mass and function that occurred with aging

**Figure 8.**
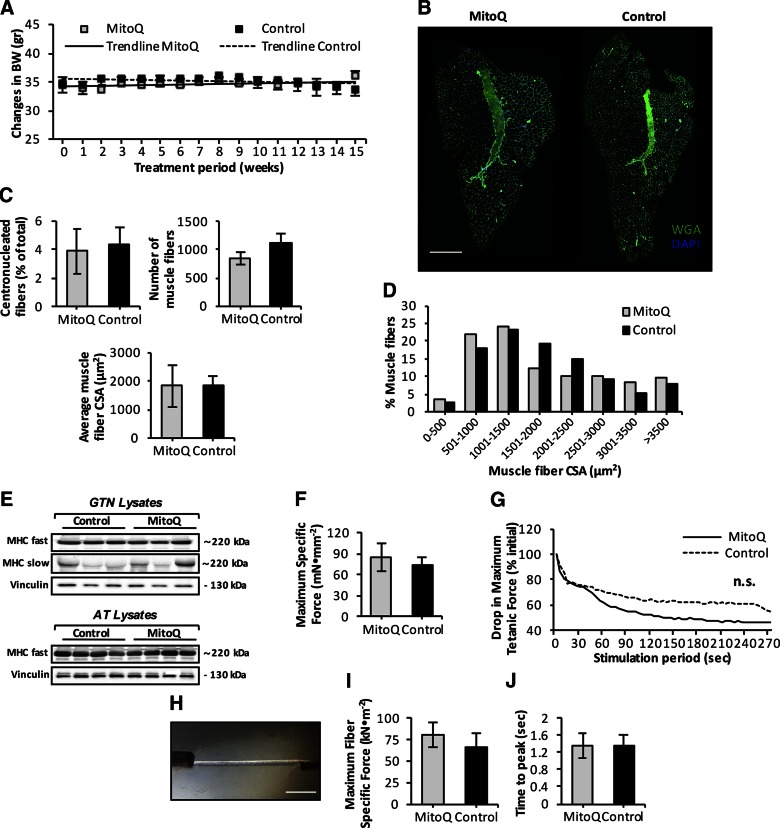
Effect of long-term mitoquinone mesylate (MitoQ) treatment on age-related loss of muscle mass and function. *A*) Time course of changes in mouse BW during 15 wk of mito-targeted MitoQ administration. Trend lines indicate no changes in BW during treatment period; BW was monitored weekly (*n* = 5–6 mice per group). *B*) Transverse sections of AT muscle from 28-mo-old control and MitoQ-treated mice obtained after 15-wk treatment period, and stained with WGA (5 μg/ml, green) to visualize extracellular matrix and DAPI (1 μg/ml, blue) to mark nuclei. Scale bar, 400 μm. *C*) Percentage of fibers showing centrally located nuclei in AT muscle of control and MitoQ-treated old mice (upper left); total number of muscle fibers in AT muscle of control and MitoQ-treated old mice (upper right); mean CSA of individual fibers from AT muscle of control and MitoQ-treated old mice (*n* = 5–6 mice per group; lower). *D*) Frequency distribution of fiber CSA of AT muscle from control and MitoQ-treated old mice (*n* = 5–6 mice per group). *E*) Representative Western blots of fast [myosin heavy chain (MHC) fast] and slow (MHC slow) MHC content in gastrocnemius (GTN, upper) and AT muscle (lower) of control and MitoQ-treated old mice. *F*) Maximum isometric specific force measured *in situ,* normalized to total fiber CSA of EDL muscle from control and MitoQ-treated old mice (*n* = 5–6 mice per group). *G*) *In situ* measurements of drop in maximum isometric specific force of EDL muscle during series of repeated isometric contractions (300 ms at 100 Hz every 5 s), expressed as percentage of initial force. Lines represent average response of 5 muscles (*n* = 5 mice per group). Although MitoQ-treated old mice showed tendency to greater decline in isometric force production during fatiguing protocol compared to controls, data were are not statistically significantly. *H*) Image of skinned myofiber isolated from AT muscle of 28-mo-old mouse attached to force transducer and high-speed length controller. Scale bar, 350 μm. *I*) *Ex vivo* measurements of maximum fiber specific force normalized to fiber CSA of skinned myofibers isolated from AT muscle of control and MitoQ-treated old mice (*n* = 25 fibers, 5 mice per group). *J*) *Ex vivo* measurements of time to peak maximum tension of skinned fibers isolated from AT muscle of control and MitoQ-treated old mice (*n* = 25 fibers, 5 mice per group).

We examined whether long-term mitoquinone mesylate treatment affected muscle mass and structure in old mice. The trend lines depicted in **Fig. 8*A*** showed no changes in BW during the 15-wk treatment. The tissue weights of several skeletal muscles and organs did not differ significantly between control and mitoquinone mesylate-treated old mice (**Table 2**), although there was a trend toward reduced spleen mass (*P* = 0.12) and increased kidney mass (*P* = 0.1). To assess changes in muscle morphology, transverse sections of AT muscle from control and mitoquinone mesylate-treated old mice were double immunolabeled with WGA (to visualize extracellular matrix) and DAPI (to mark nuclei) (Fig. 8*B*). Histologic analysis revealed no changes in number of centrally nucleated fibers (Fig. 8*C*, left), total number of fibers per AT muscle (Fig. 8*C*, right), or average muscle CSA (Fig. 8*C*, lower) in response to the mitoquinone mesylate treatment. Quantitative analysis of individual fiber CSA showed no differences in myofiber size (Fig. 8*D*) or fiber type distribution (Fig. 8*E*) of AT or gastrocnemius skeletal muscles between control and mitoquinone mesylate-treated old mice. Next, we examined the effect of mitoquinone mesylate administration on age-related changes in muscle function (5, 17) (Fig. 8*F–J*). Functional measurements of EDL muscle force production *in situ* revealed no changes in maximum isometric specific force between control and mitoquinone mesylate-treated old mice (Fig. 8*F*). *In situ* measurements of the decline in force generation by EDL muscles during a series of repeated isometric contractions revealed a trend toward a greater decline in force in mitoquinone mesylate-treated mice compared to values from control old mice, but the apparent greater decline in the mitoquinone mesylate-treated mice was not statistically different (Fig. 8*G*). Finally, to evaluate the force production at the single-fiber level, we recorded *ex vivo* muscle force of single isolated skinned fibers (Fig. 8*H*) obtained from the AT muscle. This allowed us to examine the force generated by sarcomeric proteins independent of innervation, fiber number, ATP levels, and calcium release (51). Neither specific force (Fig. 8*I*) nor the time to peak maximum tension (Fig. 8*J*) differed between the control and mitoquinone mesylate-treated old mice. Collectively, these data reveal that long-term mitoquinone mesylate treatment did not alter age-related muscle atrophy, or any of functional deficits associated with aging skeletal muscle.

**TABLE 2.** Comparison of tissue weights from control and mitoquinone mesylate-treated old mice

Tissue	MitoQ	Control
BW (g)	35.2 ± 1.4	33.6 ± 2.6
AT (mg)	43.4 ± 3.4	40.4 ± 2.9
EDL (mg)	10.1 ± 0.5	9.8 ± 0.3
Gastrocnemius (mg)	154.6 ± 9.9	139.3 ± 6.6
Soleus (mg)	9.8 ± 0.7	8.7 ± 0.4
Liver (g)	1.91 ± 0.16	1.8 ± 0.13
Spleen (mg)	134 ± 21.1	273.3 ± 72.3
Kidney (mg)	273.2 ± 25.9	226.1 ± 12.8
Heart (mg)	211.4 ± 14	195.3 ± 14.5
Lung (mg)	185.2 ± 2.5	175.3 ± 8.9
Brain (mg)	488.8 ± 4.9	468.6 ± 8.9

MitoQ, mitoquinone mesylate. Values are presented as means ± sem (*n* = 5–6 mice per group).

## DISCUSSION

Considerable evidence has indicated that skeletal muscle decline with advancing age is associated with an increased oxidative status in redox-responsive proteins (52) and increased oxidative modifications of macromolecules including DNA, proteins, and lipids (4). Altered mitochondrial redox homeostasis has been proposed to play a key role in sarcopenia (19, 21, 53), and skeletal muscle mitochondria have been reported to exhibit an age-dependent increase in mtROS (16, 17). Aging of skeletal muscle is associated with mitochondrial dysfunction including reduced maximal ATP-generating capacity, impaired function of the mitochondrial permeability transition pore, and reduced maximal respiratory capacity (21, 31). Although cumulative oxidative damage has been suggested to induce age-associated decline in mitochondrial function (24), the effect of mitochondrial dysfunction and mtROS as the underlying key regulators of the age-related atrophy process remains an area of active research (25, 26).

Skeletal muscle produces ROS from a variety of subcellular sites (14), and studies assessing the potential role of mtROS as the underlying mechanism of mitochondrial dysfunction and muscle wasting have been restricted in part by a the lack of interventions to selectively target mtROS. In the current study, we determined the time course of age-related phenotypic/structural changes that occur in skeletal muscle and assessed the contribution of mtROS by utilizing the mitochondria-targeted ubiquinone derivative mitoquinone mesylate, which may selectively protect mitochondria from oxidative damage.

Data indicate that age-related loss of muscle mass occurred after 24 mo of age in these C57BL/6 mice and was attributed to a significant reduction in fiber CSA compared to 18-mo-old mice. Our findings are in agreement with recent human studies, highlighting age-dependent fiber atrophy as a primary cause of loss of muscle mass with advancing age (22).

Previous work from our group (16) and others (17) has shown that age-dependent loss of muscle mass and function is associated with increased mitochondrial H_2_O_2_ emission and oxidative damage (21), suggesting that changes in mitochondrial redox homeostasis toward an oxidized state may be a contributor to skeletal muscle aging. To directly assess the effect of age-related changes in the mitochondrial redox environment, we used a long-term drug intervention approach with use of mitoquinone mesylate. This compound has been developed as a therapy for humans and has undergone phase 1 and 2 clinical trials (28); it comprises a ubiquinone moiety covalently attached through an aliphatic 10-carbon chain to triphenylphosphonium, a lipophilic cation that accumulates several hundred–fold within mitochondria (54, 55). Mitoquinone mesylate is absorbed/bound to the matrix-facing surface of the inner mitochondrial membrane (into the hydrophobic core of the phospholipid bilayer) (54), driven by the membrane potential, and there it is thought to be continually recycled to the active ubiquinol antioxidant by complex II of the respiratory chain (28).

We assessed the potential of mitoquinone mesylate to rescue myofiber atrophy observed in mice between 24 and 28 mo of age and provide evidence that drug treatment failed to prevent the age-related reduction in fiber CSA. We then examined the effect of long-term administration of mitoquinone mesylate to attenuate age-dependent changes in mtROS and redox homeostasis. To our surprise, mitoquinone mesylate treatment did not reduce mtROS or provide clear antioxidant protective effects; indeed, we observed an increase in MitoSOX Red oxidation in the treated mice. Recent *in vitro* studies using skeletal muscle C2C12 cells have also provided similar results; palmitate-induced ROS production was further increased in response to mitoquinone mesylate treatment (56). Thus, the lack of any substantial antioxidant effects in response to mitoquinone mesylate administration did not allow us to directly address the original question posed in the current study: whether age-dependent changes in mitochondrial redox homeostasis play a major role in age-related muscle atrophy.

Skeletal muscle of mitoquinone mesylate-treated old mice showed some alterations in the expression levels and activity of RONS regulatory systems and elevated mitophagic potential. We further addressed whether mitoquinone mesylate influenced age-related changes in mitochondrial function and muscle force. We found that long-term administration of mitoquinone mesylate did not significantly alter mitochondrial function or muscle function, although there was a tendency to adversely affect mitochondrial respiration and a decline in force during a series of repeated isometric contractions. It is noteworthy that neither of these analyses reached statistical significance. In relation to this, recent *in vitro* studies using state-of-the-art respirometer technology (Seahorse Bioscience, ‎North Billerica, MA, USA) have reported decreased mitochondrial respiration (56) and respiratory uncoupling (57) in skeletal muscle C2C12 myoblasts and endothelial cells in response to mitochondria-targeted coenzyme Q analogs.

The relative increase in MitoSOX Red oxidation indicating increased mtROS observed in response to long-term administration of mitoquinone mesylate *in vivo* may be related to the quinone group, which has previously been shown to participate in redox cycling (53, 58). Quinone-containing compounds may undergo a 1-electron reduction by flavin-containing enzymes to form semiquinone radicals, which in turn may rapidly react with O_2_ to produce superoxide (59, 60). The work presented in this study indicates that mitochondrial superoxide production, assessed *via* changes in MitoSOX Red fluorescence, was increased in response to the drug treatment. Other *in situ* and *in vitro* studies have also provided evidence that mitoquinone mesylate can augment mtROS production by complex I (58, 61), and previous work using submitochondrial particles and purified mitochondrial complex I also suggested that redox cycling of mitoquinone mesylate can occur at the 2 sites on complex I proximal and distal to the rotenone binding site (53).

Potential prooxidant effects of mitoquinone mesylate *in vivo* may also potentially be explained by the localization of its large hydrophilic core in the aqueous phase. Factors that favor superoxide production by ubiquinones are reduction to the ubiquinol and the extent to which they are present in the aqueous environment (28, 62). Deprotonation of ubiquinol, the reduced form of mitoquinone mesylate, in the aqueous phase could lead to an oxidation reaction of quinol, generating superoxide (53, 62). These potential mechanisms offer plausible explanations why the current study has indicated that long-term mitoquinone mesylate treatment enhanced mtROS production in skeletal muscle of old mice.

Previous *in vivo* studies have subjected rodents to long-term administration of mitoquinone mesylate in wild-type C57BL/6 (28) as well as a transgenic mouse model of Alzheimer disease (27) and have showed either antioxidant protective effects (27) or lack of changes in redox homeostasis (28) in brain, liver, and heart tissue. Both of these studies used young mice (4–8 wk old), and the responses of tissues to this compound may potentially change in old organisms. Alternatively, it is possible that the inherent differences in specific tissue redox potential may alter the efficacy of mitoquinone mesylate in protecting mitochondria from oxidative damage.

To our knowledge, this is the first study to utilize a long-term mitoquinone mesylate pharmacologic approach and to examine the effect on skeletal muscle mitochondrial redox homeostasis, organelle integrity, and function as well as age-related loss of muscle mass and function. Mitoquinone mesylate failed to attenuate age-related oxidative damage or rescue the sarcopenic phenotype and functional deficits associated with aging of skeletal muscle.

## Abbreviations

AT: anterior tibialis
B2M: β-2 microglobulin
BW: body weight
CS: citrate synthase
CSA: cross-sectional area
EDL: extensor digitorum longus
FCCP: protonophore carbonylcyanide-p-trifluoromethoxyphenyl hydrazone
GAPDH: glyceraldehyde 3-phosphate dehydrogenase
4-HNE: 4-hydroxynonenal
mtDNA: mitochondrial DNA
mtROS: mitochondrial reactive oxygen species
NOX4: nicotinamide adenine dinucleotide phosphate oxidase 4
3-NT: 3-nitrotyrosine
OGG1: oxoguanine DNA glycosylase
8-OHdG: 8-hydroxydeoxyguanosine
2-OH-Mito-E^+^: 2-hydroxyethidium
PGC-1α: peroxisome proliferator-activated receptor α
PINK1: PTEN-induced putative kinse 1
PRX: peroxiredoxin
PRXV: peroxiredoxin V
qRT-PCR: quantitative RT-PCR
RCI: respiratory control index
RONS: reactive oxygen and nitrogen species
ROS: reactive oxygen species
RPS29: ribosomal protein S29
SOD: superoxide dismutase
TMRM: tetramethylrhodamine, methyl ester
TRX: thioredoxin
TRXR: thioredoxin reductase
UCP: uncoupling protein
WGA: wheat germ agglutinin
